# Oral Thrombus: Report of 122 cases with clinically descriptive data

**DOI:** 10.4317/medoral.21627

**Published:** 2017-04-08

**Authors:** Priscila-Lie Tobouti, Fernanda-Mombrini Pigatti, Maria-Carolina Martins-Mussi, Suzana-Cantanhede-Orsini Machado de Sousa

**Affiliations:** 1 PhD. School of Dentistry-University of São Paulo, Oral Pathology department Av. Professor Lineu Prestes, 2227, Cidade Universitária,05508-000- São Paulo- SP- Brazil; 2 PhD. Universidade Federal do Oeste da Bahia- UFOB. Campus Reitor Edgard Santos - Prainha. Rodovia BA 827, s/n. 47800-000- Barreiras-BA-Brazil

## Abstract

**Background:**

The aim of the present study was to assess the frequency and characterize clinic-pathologic aspects of thrombus occurring as a single lesion or in association with other oral pathologies.

**Material and Methods:**

122 cases of thrombus from the oral cavity were retrieved. Information regarding site of the lesion, age, sex and clinical diagnosis or hypothesis and associated lesions were collected from the patients´ records.

**Results:**

The lesions occurred in a wide age range but the 5th decade was the most prevalent and female patients were more affected. The most frequent site for the lesion was the lip, followed by tongue, buccal mucosa, alveolar ridge, gingiva, floor of the mouth and vestibule. Thirty-five cases were associated with other vascular anomalies or actinic cheilitis. Microscopically, typical thrombus morphology was present. Organized thrombus presented neovascularization and fibroblasts, associated with hemorrhagic areas.

**Conclusions:**

Only 4 cases of oral thrombus have been described in the oral cavity. Given the limited number of cases reported, the importance of a thrombus in the oral cavity is not well established. This study contributes to establishing the profile of patients presenting oral thrombus, a lesion not rare but not well documented.

** Key words:**Oral cavity, phlebothrombosis, thrombosis.

## Introduction

Thrombosis is a multifactorial disease and multiple risk factors are prerequisite for thrombus progress. Major risk factors other than age include exogenous factors such as surgery, hospitalization, immobility, trauma, pregnancy, puerperium and hormone use. The endogenous factors comprise diseases such as overweight, malignant neoplasm, and disorders of hypercoagulation (1-3).

Venous thrombus, also called phlebothrombosis, usually presents as deep-vein thrombosis of the lower limbs and are less common in other veins (2,4). The involvement of the oral and maxillofacial region is probably not uncommon, but only four cases have been reported (5-8).

In this study, we aimed to evaluate a series of thrombus affecting the oral cavity and discussed their epidemiological and histopathological aspects.

## Material and Methods

This study was approved by the Human Research Ethics Committee of the Dental School- University of São Paulo, approval number: 754-618/2014.

All cases histologically diagnosed as thrombus at the Oral Pathology Service at the School of Dentistry, University of São Paulo, between the years 1997 and 2014, were retrieved and one hundred and twenty two (122) were included in the present study. Information regarding site of the lesion, age, sex and clinical diagnosis or hypothesis and associated lesions were collected from the patients´ records.

The slides from each case, included in this study, were reviewed by two pathologists according to the histological features described.

## Results 

**Figure 1:**
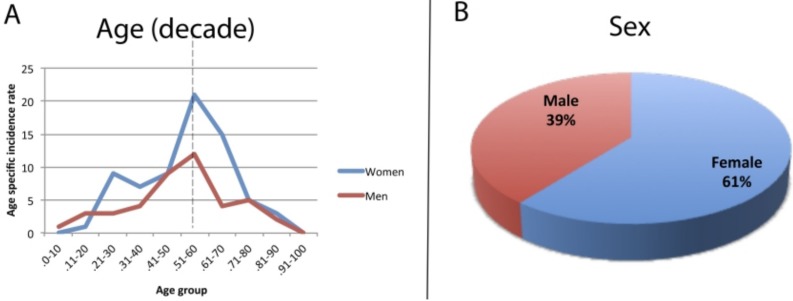
Oral thrombus clinical characteristics: (A) Age incidence in decades. Note the prevalence of the 50th decade. (B) Sex incidence: Females are more affected in a ratio of 1.56:1.

Within the 18-year period, 122 cases of oral thrombus were retrieved. The lesions occurred at any age, but the 5th decade was the most prevalent, for both men and women (Fig. 1A), with a mean age of 52 years old. Moreover, the incidence rates were slightly higher in women during childbearing years (16-44 years), compared to men of similar age (30% of the women at that age were affect and 28% of the men were affected). The higher incidence rates were after age 45 years, comprehending ~70%. Women were more affected, at a ratio of 1.56:1 (Fig. 1B).

The prevalent site of the lesion in the oral cavity was the lip (65 cases), followed by tongue (22 cases), buccal mucosa (13 cases), alveolar ridge (6 cases), gingiva (3 cases), floor of the mouth (4 cases), vestibule (3 cases) and not specified (6 cases) ( Table 1).

**Table 1:**
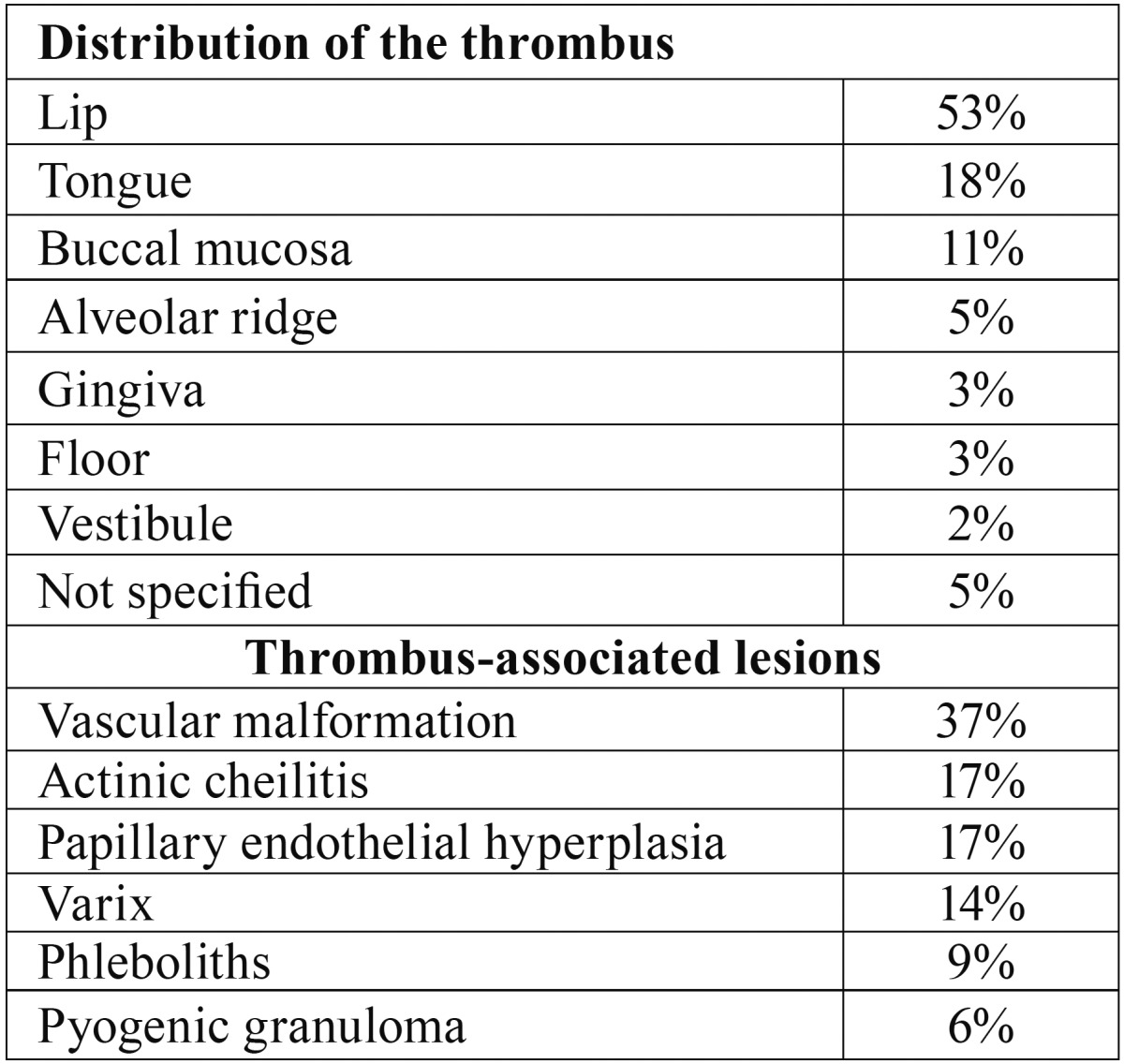
Distribution of the thrombus and prevalence of lesions associated to thrombus, in the oral cavity.

Only 5% of surgeons made a diagnosis of thrombus as the first clinical impression. Forty-seven percent of the clinical hypotheses were vascular anomalies and the most common was hemangioma (30%). Other clinical hypotheses were fibroma (14%), mucocele (14%), followed by others less mentioned such as soft tissue tumor and salivary gland lesions. Only 1% of surgeons diagnosed as malignant tumor or an infectious disease.

**Figure 2:**
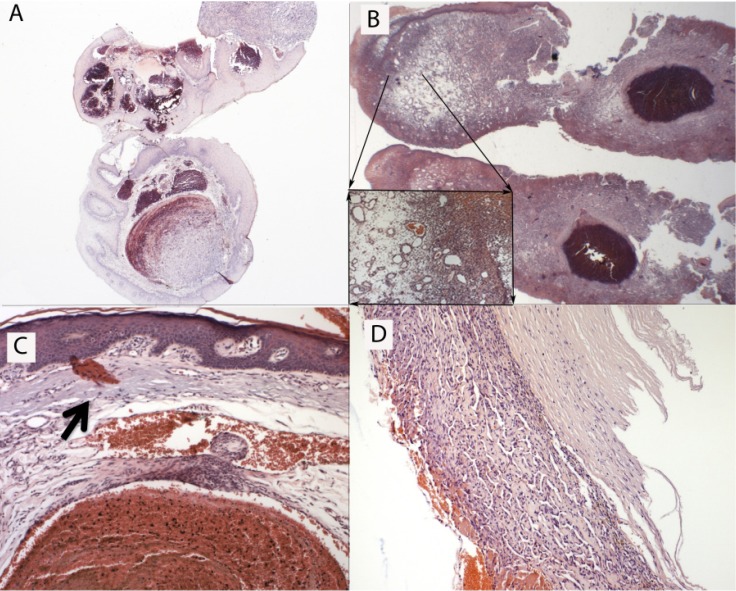
Thrombus in association with other pathologies. (A) Vascular malformation; (B) Pyogenic granuloma; (C) Actinic cheilitis, (black arrow shows solar elastosis); (D) Papillary endothelial hyperplasia.

Thirty-five cases (29%) were associated with another lesion ( Table 1), as follows: 13 with vascular malformations (37%) (Fig. 2A), six with papillary endothelial hyperplasia (PEH) (17%) (Fig. 2B), six with actinic cheilitis (17%) (Fig. 2C), five with varix (14%), three with phleboliths (9%) and two with pyogenic granuloma (6%) (Fig. 2D).

**Figure 3:**
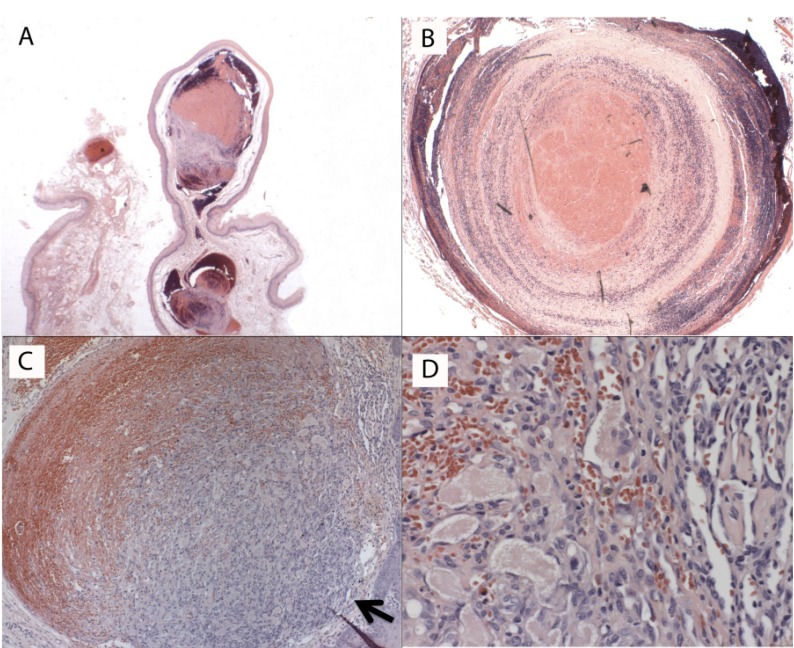
Histopathological aspects of thrombus: (A) An entire thrombus; (B) A detail of the lumen fulfilled by laminations (Zahn`s lines) and deposits of platelet and fibrin; (C) Thrombus partially attached to the vessel wall (black arrow); (D) Organized thrombus showing neovascularization.

Microscopic aspects were typical of thrombus (Fig. 3A) located in any other site of the body. These aspects mainly occurred in veins of which the lumen was partially or completely fulfilled by laminations formed by the presence of deposits of platelet and fibrin, as in the Zahn`s lines (Fig. 3B). In most cases, a focal area was attached to the vessel wall (Fig. 3C). Depending on the maturation, thrombus organization showing neovascularization (Fig. 3D) and fibroblasts could be observed. Calcifications were present in 9% of the cases (Phleboliths), and 17% of the cases showed a reactive lesion (PEH).

## Discussion

In our experience, the incidence of oral thrombosis is not rare, however the real incidence of oral thrombus is unknown. There are only 4 cases of thrombus in the oral cavity reported in the literature, in which all of the reported cases occurred in the tongue (5-8). Here, we described 122 cases of oral thrombus in which 65 occurred in the lips, 22 occurred in the tongue, 13 occurred in buccal mucosa, 6 occurred in the alveolar ridge, 4 occurred in the mouth floor, 3 occurred in the gingiva, and 3 occurred in the vestibule.

Perhaps due to the scarcity of reports showing oral thrombus, most of the clinicians did not take into account this possibility when examining their patients. However, a vascular lesion was mentioned by most of them, and the hypothesis of hemangioma was the most frequent among vascular lesions, despite the lesions` time of evolution.

The mean age of incidence was 52 years. Age is considered the strongest risk factor for thrombosis mainly due to alterations that occur with aging, such as variations in the procoagulants, body mass gain and also increase in intravascular coagulation proteins such as D-dimer and prothrombin demonstrating a hypercoaguable state (1). Another strong risk factor, in women, is puerperium, hormone therapy, oral contraception (9,10) and pregnancy (11). However, in our study, the peak of incidence was not as higher in women during childbearing years (16-44 years) than men with a similar age, as seen in deep-vein thrombosis (11,12), and still lower than the incidence rate of older women (1,13).

Microscopically, initially, the thrombus often exhibits laminations, which are platelet and fibrin deposits alternating with red cell-rich layers (Zahn’s lines). In this phase, the thrombi are focally attached to the vascular wall and no reactions between endothelium and thrombus are noted. Over time, the permeation of fibroblasts causes endothelial budding and proliferative change in the medial ring. Ultimately, the thrombus is hyalinized with central cavities, and in some cases, the neo-formed vessels with red blood cells are evident (14).

The histophysiology of a thrombus is similar in arteries and veins. All of our cases occurred in veins. The explanation for this is unknown. Possible factors for the development of a venous thrombus, are those that contribute to the stasis: fragility of the vessels, increased coagulation factor levels, damage in the function of the venous valves and lower function of the anticoagulants (1).

The main cause of development for a thrombus could be one or more of the components that Virchow described in 1856. The Virchow`s triad, as it is called, is composed of 1. endothelial injury; 2. abnormal blood flow and 3. Hypercoagulation (15). Arterial or cardiac thrombi usually begin after a rupture of an atherosclerotic plaque and at sites of turbulence or endothelial injury, whereas venous thrombi characteristically occur at sites of stasis and hypercoagulability (16). Thrombus could develop because of a pre-existing lesion or syndrome such as varicose (varix) (17) and antiphospholipid (6), respectively. In the present cases an association with vascular anomalies and actinic cheilitis was found in some cases.

When associated with vascular anomalies, thrombus formation in the oral cavity is likely to be caused by the presence of tortuous vessels, which can disturb the normal blood circulation leading to the adherence and aggregation of the platelets, even without vessel injury, hypercoagulability or trauma (16,18). For instance, in the cases studied, association with varicose veins was observed in five cases. A varicose vein is the main cause of superficial vein thrombosis (17,19). There are both reflux and dysfunction of the valves, as well as dilatation, caused by an increase of the venous pressure, which causes functional changes in the vein wall. This tension increases the metalloproteinase activity, which can affect the structural integrity of the vein wall (20). In the cases associated with actinic cheilitis, the chronic physical exposure to the sun leads to a degeneration of collagen and elastic fibers (21,22). This aggression could eventually cause an alteration of the walls of the vein. But, it is difficult to affirm that the thrombus was secondary to the actinic cheilitis because actinic cheilitis occurred in only 6 of the 122 cases, with no other cases reported in the literature. On the other hand, the other lesions associated with thrombus in the present study are more likely to be secondary to the thrombus, such as PEH, a non-neoplastic lesion of unclear pathogenesis (23-25) and phleboliths (26).

Oral thrombus is not a rare lesion, but it is not well reported, probably due to the low risk of embolization and mortality. To our knowledge, there is no report showing embolization of an oral thrombus. However, even superficial vein thrombosis can undergo a venous thromboembolism (11,17).

In conclusion, this study contributes to the establishment of the profile of patients affected by oral thrombus and its association with other entities. Moreover, our results open new avenues for future studies in the causes and possible consequences of an oral thrombus.
